# Laparoscopic Repair of Transmesosigmoid Hernia Following Robot-Assisted Abdominoperineal Resection: A Case Report

**DOI:** 10.7759/cureus.86061

**Published:** 2025-06-15

**Authors:** Hiroshi Saito, Masanori Kotake, Kaeko Oyama, Takuo Hara, Noriyuki Inaki

**Affiliations:** 1 Department of Surgery, Koseiren Takaoka Hospital, Takaoka, JPN; 2 Department of Gastrointestinal Surgery/Breast Surgery, Graduate School of Medical Science, Kanazawa University, Kanazawa, JPN

**Keywords:** colorectal cancer, ileus, internal hernia, laparoscopic surgery, transmesosigmoid hernia

## Abstract

Sigmoid mesocolon hernia is a rare type of internal hernia that can rapidly progress to vascular compromise, necrosis, and intestinal perforation. Therefore, rapid diagnosis and surgical treatment are important. We present a case of transmesosigmoid hernia following robot-assisted abdominoperineal resection that was successfully treated using a laparoscopic approach.

An 80-year-old woman underwent robot-assisted abdominoperineal resection for anal canal cancer. The postoperative course was uneventful. Two months after the surgery, the patient presented with upper abdominal pain. Computed tomography revealed small bowel obstruction. We diagnosed the patient with strangulating intestinal obstruction and performed emergency laparoscopic surgery. Intraoperative findings revealed small intestinal strangulation and herniation through a defect in the sigmoid mesocolon. The strangulated intestine was released, and the defect was closed using barbed sutures. The patient was discharged on postoperative day 9 without complications. Intraoperative video from the previous surgery showed a small defect in the sigmoid mesocolon suspected to be the origin of the transmesosigmoid hernia. The defect may have formed during the medial approach using an electric scalpel.

We emphasized the need for intraoperative vigilance when using energy devices during mesenteric dissection and any mesenteric defect created during surgical procedures should be promptly closed to prevent subsequent transmesenteric internal hernias.

## Introduction

Internal hernia rarely causes small bowel obstruction, with a reported incidence of up to 5.8% of intestinal obstruction [1-3]. Rapid diagnosis and surgical treatment are crucial because the mortality rate exceeds 50% when strangulation occurs [4,5]. Sigmoid mesocolon hernia is particularly rare, with transmesosigmoid hernia representing a subtype of this group [6]. Moreover, there have been no reported cases of sigmoid mesocolon hernia occurring after robotic surgery to date. We report a rare case of transmesosigmoid hernia following robot-assisted surgery to highlight the importance of intraoperative defect closure and the efficacy of laparoscopic management.

## Case presentation

An 80-year-old female patient was referred to our hospital for melena that had persisted for three months. A detailed examination revealed anal canal cancer. Robot-assisted abdominoperineal resection was performed as a radical treatment option. The left colic artery was preserved during the surgery. The postoperative course was uneventful, and the patient was discharged on postoperative day 13. Pathological evaluation revealed a ulcerative and infiltrative-type tumor of the anal canal, with a pathological classification of T3N0M0 stage II. Postoperative follow-up without adjuvant treatment was planned.

**Figure 1:**
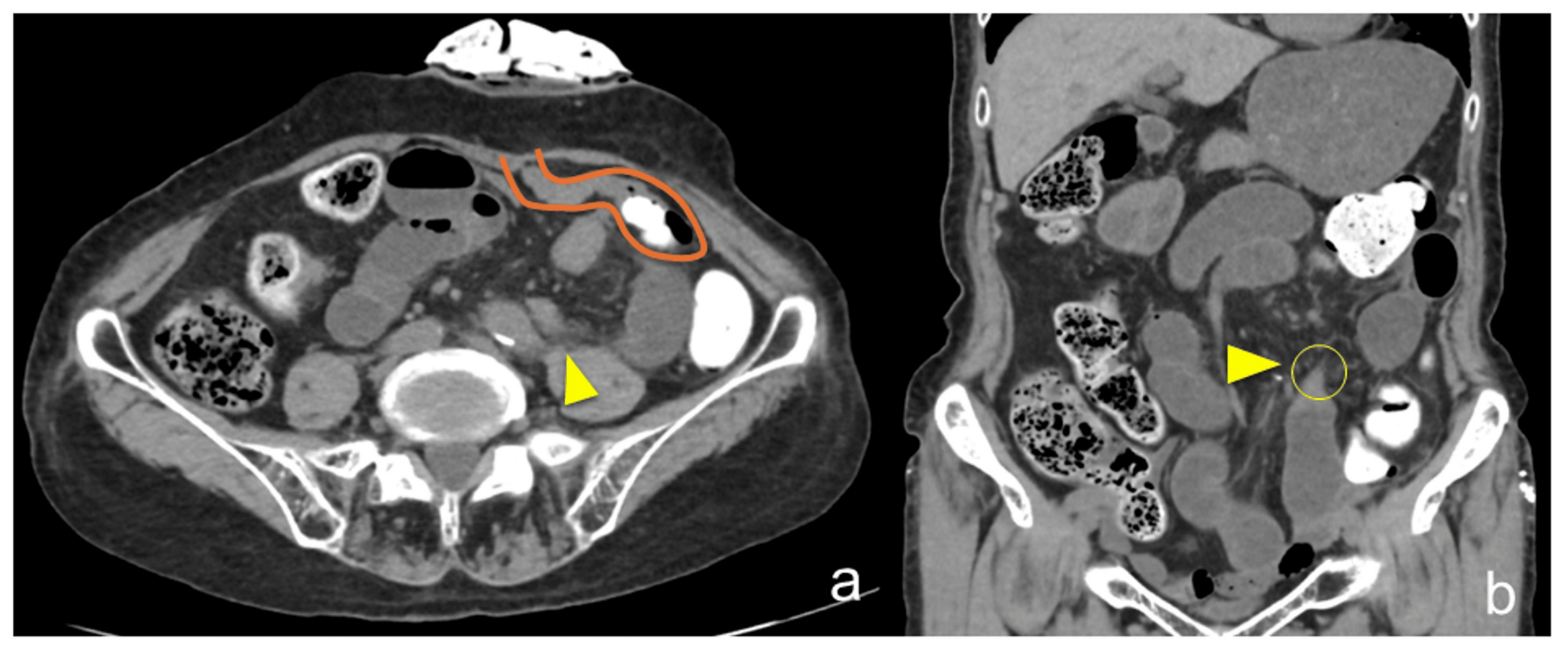
CT findings. a. Axial CT showing a sigmoid stoma in the left lower abdomen with a transition point (arrowhead) indicating small bowel obstruction. b. Coronal CT demonstrating a beak sign (arrowhead) at the site of obstruction, suggestive of a closed-loop obstruction. CT: computed tomography

**Figure 2:**
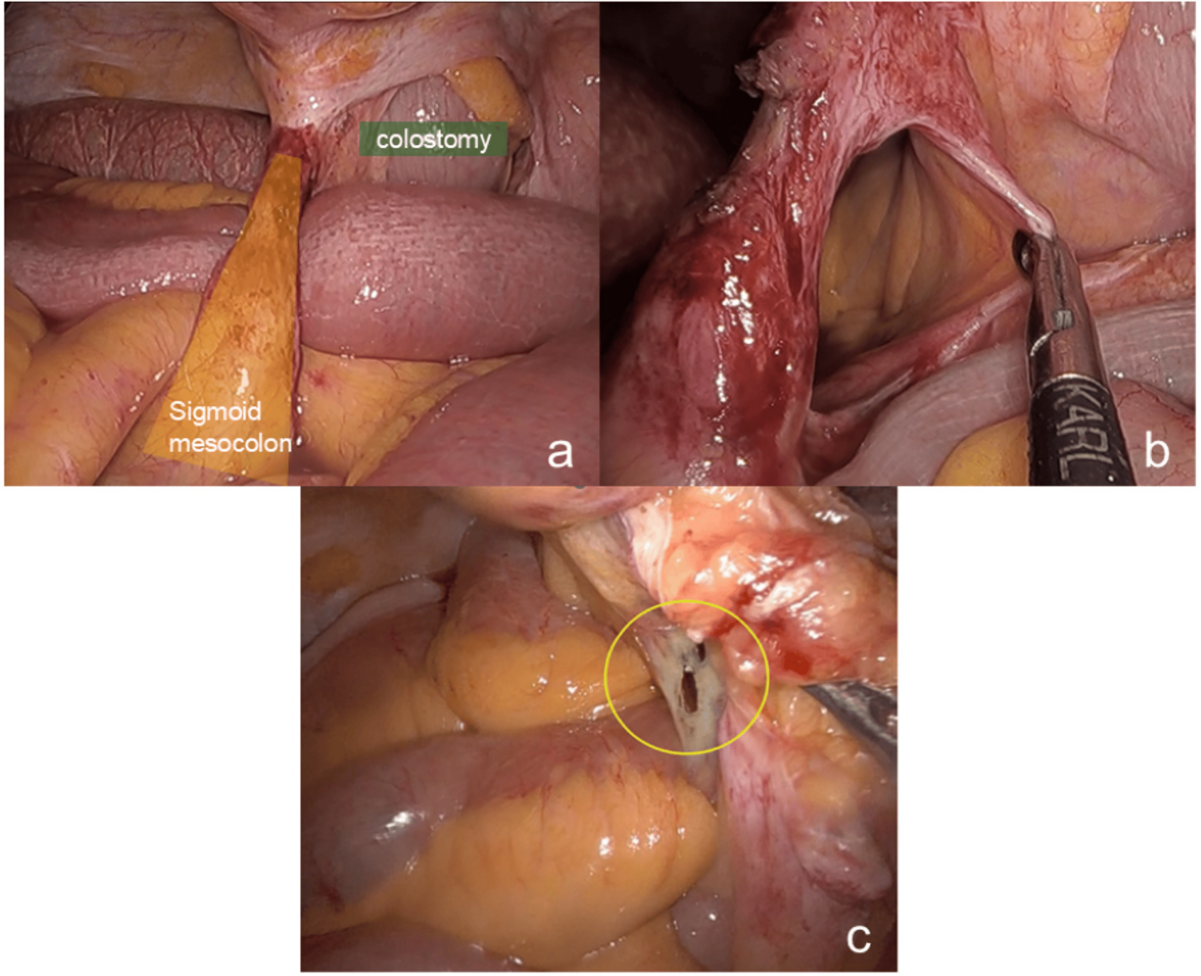
Laparoscopic findings of the mesosigmoid hernia. a. The small intestine is incarcerated within the orifice of the mesosigmoid hernia. b. The defect size measures approximately 3 cm. c. Retrospective review of the robotic surgery video revealed a small defect in the sigmoid mesocolon, likely the origin of the hernia, which went undetected at the time of the initial surgery.

The patient presented to the outpatient clinic with upper abdominal pain two months post-surgery. Nausea was present, but there was no fever, and a small amount of stool was observed from the stoma. Physical examination identified a point of moderate tenderness in the upper abdomen. There were no significant findings in the laboratory data, including normal levels of white blood cells, C-reactive protein (CRP), and lactate. Computed tomography (CT) revealed a closed-loop obstruction with a beak sign at the transition point near the sigmoid colostomy, with proximal jejunal dilation and no free air or fluid collection(Figure 1). We diagnosed the patient with a strangulating intestinal obstruction due to the previous surgery. Emergency laparoscopic repair was performed. The patient was placed in a supine position, and general anesthesia was administered. The first port of the scope was inserted via an open technique on the right side of the abdomen, followed by the insertion of two additional ports. Intraoperative findings revealed a sigmoid stoma in the left lower abdomen, with no adhesions. There was a defect in the sigmoid mesentery, with an incarcerated small intestine in the defect (Figure 2a, 2b). Intraoperative diagnosis confirmed a transmesosigmoid hernia as the cause of the obstruction. The incarcerated bowel was released, and the defect was closed with 3-0 non-absorbable barbed sutures to prevent future transmesenteric internal herniation. As the small intestine was viable, no intestinal resection was required.

The postoperative course was uneventful, and the patient was discharged on postoperative day 9. We reviewed the intraoperative video from the previous surgery and found a small defect in the sigmoid mesocolon suspected to be the origin of the transmesosigmoid hernia (Figure 2c). 

Three years after the initial surgery, the patient died due to another cancer. During the clinical course, neither recurrence of the anal canal cancer nor any surgery-related complications were observed.

## Discussion

This case report aims to discuss the management and outcomes of transmesosigmoid hernia, a postoperative complication. Internal hernias are caused by defects within the boundaries of the peritoneal cavity. These defects may be congenital, traumatic, operative, post-inflammatory, or idiopathic in origin. The autopsy incidence of internal hernias ranges from 0.2% to 2%, with most being asymptomatic and acquired [1,3,7,8]. Sigmoid mesocolon hernia is rare, accounting for approximately only 5% of all internal hernias [6,7,9].

Bensons and Killen [6] classified sigmoid mesocolon hernias into three types: The first type is the intersigmoid hernia, which involves herniation into the intersigmoid fossa located at the lateral attachment of the sigmoid mesocolon. This fossa forms during the fusion of the left peritoneal surface of the sigmoid mesentery with the parietal peritoneum of the posterior abdominal wall, forming the line of Toldt. The second type is the transmesosigmoid hernia, which is characterized by the incarceration of intestinal loops through a full-thickness defect in the sigmoid mesocolon. The third type is the intramesosigmoid hernia, which is caused by a congenital, oval defect unrelated to the intersigmoid fossa and located in juxtaposition with the colon. The hernial defects involve only one leaf of the sigmoid mesocolon. In this case, we diagnosed a transmesosigmoid hernia likely caused by previous surgery, as confirmed by reviewing the intraoperative video. With respect to internal hernias following robot-assisted surgery, cases of transmesocolic hernias after pancreaticoduodenectomy have been documented [10]. However, to the best of our knowledge, no such cases have been reported following rectal surgery.

Preoperative diagnosis of an internal hernia is uncommon, and predicting the specific types of internal herniation is even more challenging. Nonetheless, the diagnosis should be considered regardless of whether there is a history of prior abdominal surgeries or external hernias. In this case, based on the patient's surgical history and the CT findings showing a closed-loop obstruction and beak sign, a diagnosis of strangulated small bowel obstruction was suspected. However, internal hernia was not initially anticipated as the underlying cause.

Laparoscopic surgery has recently been introduced as a minimally invasive procedure for managing patients with intestinal obstruction. Compared with laparotomy, laparoscopic surgery offers several advantages, including reduced postoperative pain, shorter hospital stay, quicker return to daily activities, fewer wound-related complications, and a lower recurrence rate of adhesions [11]. Additionally, laparoscopy serves as both a diagnostic and therapeutic tool, particularly in cases that are difficult to diagnose preoperatively or in patients with suspected strangulated intestinal obstruction, thereby avoiding unnecessary laparotomies. In the present case, the patient's symptoms were not severe, vital signs were stable, and preoperative imaging suggested intestinal strangulation. Therefore, we opted for laparoscopic surgery. Owing to the use of robotic surgery, no intra-abdominal adhesions were observed, and a safe operation with a clear view was performed. To identify the cause of the mesocolonic defect, we observed the operative video of the initial surgery after the secondary surgery and confirmed a small hole in the sigmoid mesocolon. Retrospectively, the defect may have formed during the medial approach using an electric scalpel and was not detected during the initial procedure. The hole likely enlarged gradually postoperatively, eventually leading to the small intestine becoming incarcerated in the hole. This case highlights the need for intraoperative vigilance when using energy devices during mesenteric dissection. Intraoperative video review may also serve as a valuable tool for quality assurance and surgical education. A key lesson from this case is that any defect in the mesocolon, even if small, should be closed intraoperatively to prevent subsequent transmesenteric internal hernias. It is also necessary to thoroughly inspect for any potential injury sites after completing all procedures.

## Conclusions

We encountered a case of transmesosigmoid hernia following robot-assisted abdominoperineal resection caused by a defect in the mesosigmoid. When utilizing energy devices during mesenteric dissection, meticulous intraoperative observation is crucial to minimize the risk of tissue injury. Furthermore, intraoperative video recordings can serve as an effective tool for surgical education. Also, any mesenteric defect created during surgical procedures, regardless of its size, should be closed to prevent the occurrence of subsequent transmesenteric internal hernias.
